# Direct sequencing of β-globin gene reveals a rare combination of two exonic and two intronic variants in a β-thalassemia major patient: a case report

**DOI:** 10.1186/s13256-022-03605-2

**Published:** 2022-10-09

**Authors:** Waseem Chauhan, Rafat Fatma, Zeeba Zaka-ur-Rab, Mohammad Afzal

**Affiliations:** 1 Human Genetics and Toxicology Laboratory, Department of Zoology, Aligarh Muslim University, Aligarh, India; 2 Department to Paediatrics, JNMC, Aligarh Muslim University, Aligarh, India

**Keywords:** β-thalassemia, Case report, CD 41/42 (-CTTT), IVS II-666 (C>T), Compound heterozygous, Silent mutation

## Abstract

**Background:**

Due to indels in the β-globin gene, patients with β-thalassemia major exhibit a range of severity, with genotype β^0^β^0^ > β^0^β^+^ > β^+^β^+^, according to the production level of the β-globin chain. More than 300 mutations have been identified in the β-globin gene.

**Case presentation:**

In this case study, we report a compound heterozygous condition with a rare concoction of four different variants (CD 3(T > C), CD41/42 (-CTTT), IVS II-16 (G > C), and IVS II-666 (C > T) in a single β-globin gene. A regular transfusion-dependent 4-year-old male patient from India was included in the study. Augmented direct sequencing of the β-globin gene helped reveal the presence of an unusual combination of different variants in a single gene. This patient clinically presented as β-thalassemia major and was genotypically considered as β^0^β^+^, although CD41/42(-CTTT) was the only causative/pathogenic mutation in the disease severity.

**Conclusion:**

Although CD41/42-(CTTT) is the only pathogenic variant among the four variants, the clinical complications of such a combination of variants (pathogenic and benign) is not well understood. Intronic mutations may have the ability to modify clinical characteristics. The variants must therefore be reclassified using additional mRNA splicing and expression-based studies. Additionally, these types of combinations may have significance in studying population migration around the world.

## Background

Tetrameric hemoglobin molecules are involved in gaseous exchange in red blood cells. The β-globin chains, when paired with the α-globin chains, result in the formation of hemoglobin tetramers. It has come to light that mutations in the β-globin gene cause either insufficient (β^+^) or halted development (β^0^), resulting in β-thalassemia major. The possible genotypes responsible for β-thalassemia major, in order of severity, are β^0^β^0^ > β^0^β^+^ > β^+^β^+^ 1.

Various studies have identified more than 300 mutations to date (Ithanet and HbVar databases). The Mediterranean region, the Indian subcontinent, Southeast Asia, and the Middle East are all home to these mutations 2, and thousands of people of various ethnicities are affected by this autosomal recessive syndrome 3. The advent of direct or Sanger DNA sequencing has increased the study of mutations in various diseases and β-thalassemia. Here, we present a case of a rare compound heterozygous condition involving four variants in the β-globin gene identified by direct sequencing. We discovered this patient with four separate variants in the β-globin gene (CD 3 [T>C], CD 41/42 [-CTTT], IVS II-16 [G>C], and IVS II-666 [C>T]) during a screening of variants among β-thalassemia major patients registered at the JNMC hospital, Aligarh, India.

## Case presentation

**Fig. 1:**
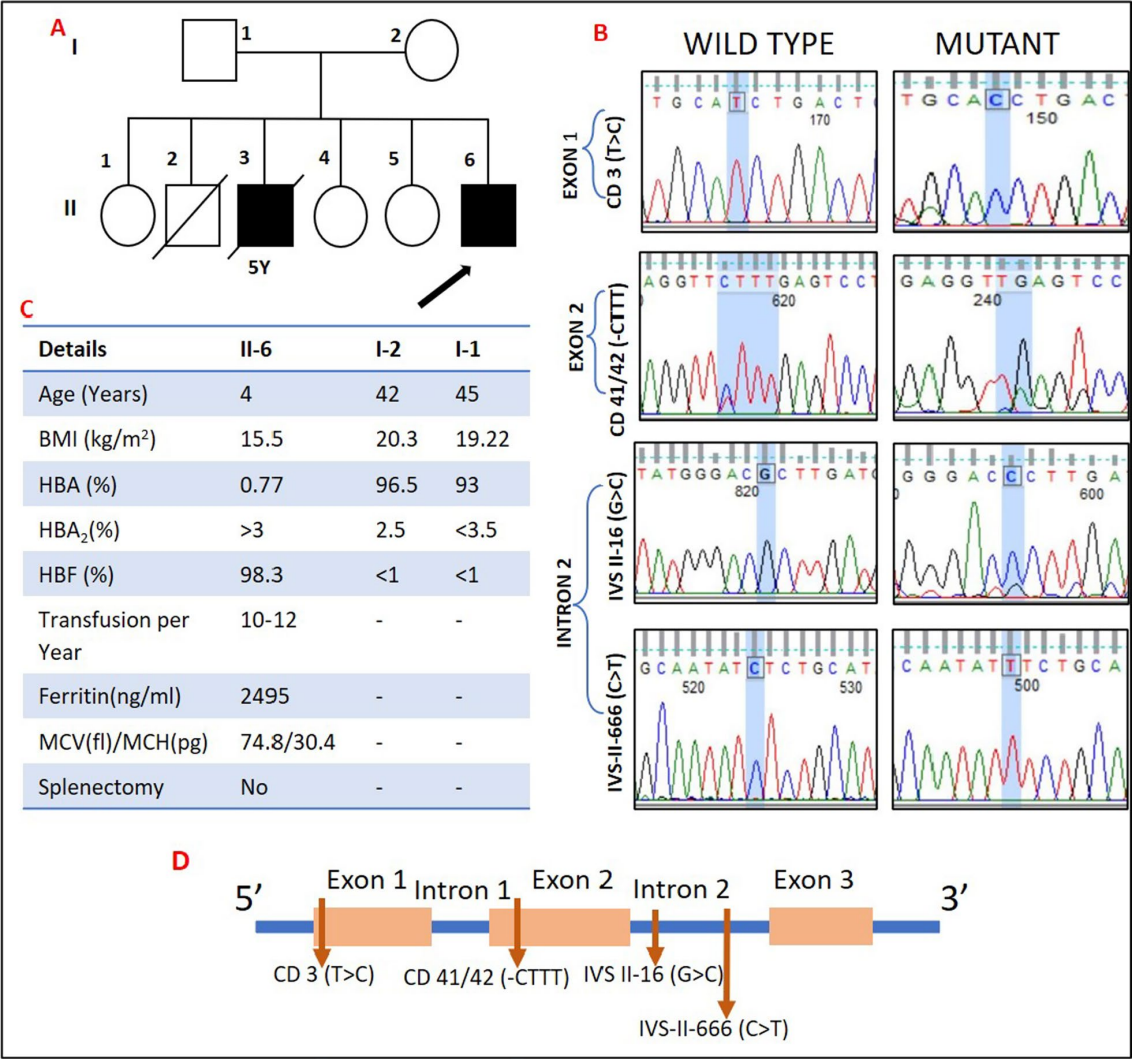
Details related to the case study. **A** The pedigree diagram illustrates family history: one male sibling (deceased) was affected with β-thalassemia major and other siblings were unaffected. **B** Sequencing data shows wild type and mutant chromatograms for both exonic (CD3 [T>C] and CD41/42 [-CTTT]), and intronic (IVS II-16 [G>C] and IVS II-666 [C>T]) variants. **C** Clinical data collected from the hospital. **D** Schematic diagram of HBB gene structure with four identified variants at their respective locations

A 4-year-old Indian boy from a low-income family (farmer) was admitted to the hospital for a routine blood transfusion. His weight and height was between the 10th and 50th centile, with −1.71 and −1.54 *Z*-scores, respectively. He had O (+ve) blood group and required 10–12 blood transfusions a year. His adult hemoglobin (HbA_**2**_) was > 3%, mean corpuscular volume (MCV) was 74.89 fl, and mean corpuscular hemoglobin (MCH) was 30.4 pg according to his medical records, and his physical examination revealed that he had knee pain and an enlarged spleen (> 3 cm below costal margin). As a result, clinicians discovered that he had β-thalassemia major and he was on folic acid 2.5 mg/day and deferasirox 30 mg/kg/day. After reviewing his family history, it was discovered that one of his two male siblings (who died) had β-thalassemia, while all of his sisters were well (Fig. 1). The parents gave their consent for the patient, but the parents and their healthy offspring declined to participate, so we removed them from the study. Medical details and blood samples were collected from the hospital once written informed consent was given. The genomic DNA was isolated according to the manufacturer’s instructions using the gDNA Blood Mini Kit (Chromous Biotech, Bengaluru, India). Additional genomic analysis was carried out according to [4]. The BLAST method in the National Center for Biotechnology Information (NCBI) database was used to confirm the identification of the collected sequences. By comparing mutations to reference sequences, direct counting was used to determine their presence (NG 000007.3 *Homo sapiens* chromosome 11). The dbSNP, Clinvar, Ithanet, and HbVar databases were then used to look for mutations that had been identified in other populations [5].

## Discussion

The patient, from western Uttar Pradesh in India, has a unique combination of four different mutations in a single gene (β-globin), along with typical developmental milestones, although he had a lower body mass index (BMI) (10–50th centile, −1.71 *Z*-score), splenomegaly, and required 10–12 routine transfusions annually. Consequently, this patient falls into the category of “severe,” according to the Mahidol severity score [6].

Since it is so close to New Delhi, India’s capital city, the western part of Uttar Pradesh has a special geographical location. As a result, this city is referred to as the national capital region (NCR), and it has attracted people of various races and ethnic groups from various Indian states. A national meta-analysis found that 52 mutations account for 97.5% of all β-thalassemia alleles, with IVS I-5 (G>C) being the most common (54.7%). IVS I-5 (G>C), IVS I-1 (G>T), 619-bp del, CD41/42 (-TCTT), and CD 8/9 (+G) accounted for 90% of all mutations among the 52 mutations [7, 8, 9]. We describe a case with an unusual combination of four distinct mutations in the β-globin gene (CD3 [T>C], CD41/42 [-CTTT], IVS II-16 [G>C], and IVS II-666 [C>T]) in our case study (Table 1, Fig. 1).

**Table 1:** All the variants, with their Human Genome Variation Society (HGVS) nomenclature, present in this rare compound heterozygous condition

Variants/SNV	HGVS nomenclature	Position on gene	Amino acid substitution	Ref. allele frequency (global)^a^	Clinical significance^b^
CD 3 (T>C); rs713040	HBB:c.9T>C	Exon 1	Histidine>Histidine	0.16030	Benign
CD 41/42 (-CTTT); rs80356821	HBB:c.126_129delCTTT	Exon 2	Frameshift (F42fs)	0.000278	Pathogenic (premature termination codon)
IVS-II-16 (G>C); rs10768683	HBB:c.315 + 16G>C	Intron 2	Intronic Variant	0.16670	Likely-benign, Benign
IVS-II-666 (C>T); rs1609812	HBB:c.316 − 185C>T	Intron 2	Intronic Variant	0.169423	Benign

Data from dbSNP^a^ and ClinVar^b^ were used for reference allele frequency (global) and clinical significance, respectively

The CD3 (T>C), rs713040 is a clinically benign variant (Clinvar), in which the codon CAC is changed from CAT and amino acid histidine is substituted for amino acid histidine, resulting in no change in the protein sequence (Table 1). However, in India, this variant is one of the most uncommon, although it is reported in Odisha Province, and in the Bangladeshi population [10, 11]. The CD41/42 (-CTTT) mutation (rs80356821) in exon 2 of the β-globin gene is a four-nucleotide deletion that induces frameshift and stops the development of β-globin protein, and was first reported in an Asian Indian with β^0^-thalassemia [7]. Clinically pathogenic codon 41/42 (-CTTT) mutations lead to significantly higher HbA_2_ levels (> 4.0) and lower MCV and MCH levels [4]. Another two variants, IVS II-16 (G>C), and IVS II-666 (C>T), were found in the intron 2 region of the gene. Previously, IVS II-16 (G>C) and IVS II-666 (C>T) have been recognized by AvaII and SspI, respectively [12, 13]. Both the variants have been reported in many previous studies from East India, Bangladesh, Saudi Arabia, Egypt, and Palestine [11, 12, 14, 15, 16, 17, 18, 19, 20, 21]. However, these polymorphisms are not involved in the gene expression and are assumed to be benign. However, when these benign variants are found in association with HbS, they act as likely pathogenic variants and convert the HbS phenotype to transfusion-dependent Hb S/β^+^-thal [22, 23]. Another report from Turkey suggests that IVS II-16 (G>C), along with IVS II-74 (T>G), are “likely pathogenic” polymorphisms because patients with this polymorphism exhibit low levels of MCV and HbA [24]. Thus, it can be hypothesized that the IVS II-16 and IVS II-666 variants may be exerting a modulatory effect by possibly affecting mRNA splicing, depending on their proximity to exon-intron boundary, and should not be regarded as innocuous. However, more next generation/whole genome sequencing research may be helpful to overcome the ambiguity, and to evaluate the true clinical significance of these variants [25]. Additionally, inheritance of such benign variants may have significance in tracing the demographic migration of humans over time [26, 27].

In this report, the child patient was affected with β-thalassemia major, having low levels of MCH and MCV. HbA_2_ was > 3.0 % and CD41/42 (-CTTT) is the only apparent pathogenic variant that causes ineffective erythropoiesis and hemolytic anemia (Ithanet).

## Conclusion

Direct gene sequencing helps in the detection of unknown and unusual gene mutations. Although all four variants (CD3 [T>C], CD41/42 [-CTTT], IVS II-16 [G>C], and IVS II-666 [C>T] have been identified in several studies, this is the first study to describe an uncommon combination (a rare compound heterozygous condition) of four distinct changes in a single gene. CD41/42 (-CTTT) is the only well-established pathogenic mutation that causes deleterious phenotypes (β^0^). The other three variants are considered as benign polymorphisms, but some reports suggest that IVS II-16 (G>C) is likely a pathogenic variant. A large number of mutations identified in the population cohorts were also included in the natural selection study. These sorts of variants (harmless and dangerous) in combination may play a significant role in population migration studies. As a result, the family histories of people who have unusual sets of variants may be useful in the study of human migration.

## Data Availability

Not applicable.

## Abbreviations

IVS: Intervening sequence
HBB: Beta hemoglobin
CD: Codon
BMI: Body mass index
HbF: Fetal hemoglobin
HbA: Adult hemoglobin
MCV: Mean corpuscular volume
MCH: Mean corpuscular hemoglobin
CBC: Complete blood count
